# The Effect of Septorhinoplasty on Psychopathology and Quality of Life

**DOI:** 10.5152/eurasianjmed.2026.251146

**Published:** 2026-02-27

**Authors:** Pınar Tekin, Yüksel Toplu, Lale Gönenir Erbay

**Affiliations:** 1 Department of Otolaryngology, Malatya Training and Research Hospital, Malatya, Türkiye; 2 Department of Otolaryngology, Malatya Park Hospital, Malatya, Türkiye; 3 Lale Erbay Psychiatry Clinic, Ankara, Türkiye

**Keywords:** Anxiety, depression, patient satisfaction, quality of life, rhinoplasty

## Abstract

**Background:**

The purpose was to assess the impact of septorhinoplasty on psychopathology and well-being and to demonstrate the effectiveness of septorhinoplasty and emphasize the importance of considering psychopathological status when selecting patients.

**Methods:**

Seventy patients scheduled for septorhinoplasty were evaluated prospectively. Following patient selection, participants were asked to complete a sociodemographic questionnaire, the Beck Anxiety Inventory (BAI), the Beck Depression Inventory, the Nasal Obstruction Symptom Evaluation Scale (NOSE), the Short Form-36 (SF-36), and the Rhinoplasty Outcome Evaluation Questionnaire (ROE) preoperatively and at 6 months postoperatively.

**Results:**

The mean age in the study was calculated as 26.14 ± 6.80. The postoperative NOSE and BAI scores of the patients decrease, while ROE score increases (*P* < .05). An increase in physical role, physical function, general health, and social functioning scores was also found in SF-36 subscales (*P* < .05). There was an inverse correlation between preoperative depression levels and postoperative satisfaction.

**Conclusion:**

Septorhinoplasty has a positive effect on quality of life and anxiety. When selecting a patient, attention should be paid to the level of depression.

**Main Points**

- Septorhinoplasty offers both functional and psychological benefits.
- A notable increase in quality of life scores was observed following surgery.
- Depression levels may influence surgical outcomes.

## Introduction

The nose is a vital organ, not only because of its respiratory function but also due to its aesthetic significance, as it occupies a central position on the face. In addition to these roles, the nose contributes to thermoregulation, serves as a sensory organ, filters and humidifies inspired air, participates in reflex mechanisms, facilitates speech and phonation, and plays a role as a secondary sexual characteristic.^1^^,^^2^ Septorhinoplasty, a cosmetic and functional nose surgery that has gained popularity in recent years, is designed to enhance nasal functions while producing predictable changes in the nose. To achieve successful outcomes in septorhinoplasty, a holistic approach to patient evaluation is required, encompassing anatomical, physiological, and psychological aspects.^3^ In addition to improving nasal respiratory function, septorhinoplasty has a significant impact on patients’ psychological well-being and overall quality of life.^2^

Quality of life is a general term used to evaluate physical, psychological, social, economic, cognitive, and sexual dimensions of life. Different methods have been used in quality of life investigation. The fact that there are many quality of life scales and no consensus scale is available makes standardization in research difficult.^4^ Quality of life forms can be general or disease specific.^5^^-^^9^

Therefore, this prospective study aimed to estimate the effects of septorhinoplasty on psychological status and quality of life by comparing preoperative and postoperative patient-reported outcome measures.

## Material and Methods

This prospective observational study included 70 patients who visited İnönü University Turgut Özal Medical Center Otolaryngology clinic because of nasal congestion and dissatisfaction with the shape of their noses, all of whom underwent functional septorhinoplasty performed by the same surgeon between 2015 and 2016. The study included participants who met these criteria: patients who were scheduled to undergo septorhinoplasty, aged 18 years or older, and signed a consent form. Exclusion criteria included psychiatric diagnosis, previous septorhinoplasty, and a condition that prevents the patient from receiving anesthesia, such as infection or bleeding diathesis. All patients underwent open septorhinoplasty.

Preoperative psychiatric screening was carried out by the xxx Psychiatry Clinic using the Structured Clinical Interview for DSM-IV Axis I Disorders (SCID-I), as defined by the Diagnostic and Statistical Manual of Mental Disorders, Fourth Edition (DSM-IV). The study sample consisted solely of individuals without a DSM-IV–defined psychiatric diagnosis.

Sociodemographic information was gathered through a questionnaire designed by the investigators (Table 1).

The Beck Depression Inventory (BDI), the Beck Anxiety Inventory (BAI), the Short Form-36 (SF-36), the Rhinoplasty Outcome Evaluation Questionnaire (ROE), and the Nasal Obstruction Symptom Evaluation Scale (NOSE) were administered to the patients preoperatively and in the sixth month postoperatively.

Structured Clinical Interview for DSM-IV Axis I, developed by Spitzer et al,^10^ is a clinical interview conducted by the interviewer in the investigation of Axis I psychiatric disorder diagnosis according to DSM-IV. This interview is conducted by a trained interviewer with individuals over the age of 18 who have the cognitive ability to continue the structured interview and who do not have agitation or severe psychotic symptoms. Adaptation and reliability assessments of the Turkish version were performed.^11^

The SF-36 was improved and utilized to evaluate quality of life. This is a self-assessment scale. It consists of 36 parts that measure 8 dimensions. The SF-36 evaluates social function, physical function, role limitations due to physical problems, emotional role, emotional well-being, bodily pain, vitality, and general health perception. The last 4 weeks of the study were evaluated using this scale. Scoring was done between 0 and 100.^12^^,^^13^

The BAI, developed in 1988, is a 21-item self-measure scale used to assess the level of anxiety symptoms, with items scored on a 4-point scale (0-3). The BDI, developed in 1961, is a self-reported measure assessing the level of depressive symptoms, with total scores ranging from 0 to 63. The Turkish versions of both inventories have been shown to be valid and reliable.^14^^,^^15^

The NOSE scale is a disease-specific instrument used to evaluate the severity of nasal obstruction symptoms. It consists of 5 items scored from 0 to 4, with higher scores indicating greater symptom severity.^16^ The ROE questionnaire is a rhinoplasty-specific outcome measure assessing both aesthetic and functional satisfaction. It includes 6 items scored from 0 to 4, with higher scores reflecting greater satisfaction.^16^

The research was reviewed and approved by the Clinical Research Ethics Committee of the İnönü University of Medicine (Decision no: 2015/208, approval date: August 16, 2015). Written informed consent was obtained from all participants.

### Statistics

All data were analyzed using the SPSS, version 17.0. Descriptive statistical measures, including percentages, means, and standard deviations, were obtained. A threshold of *P* < .05 was used to determine statistical significance. Nonparametric tests were employed since the Kolmogorov–Smirnov test indicated a non-normal distribution of the variables (*P* < .05). The Wilcoxon signed-rank test was applied for preoperative and postoperative comparisons. Comparisons between 2 independent groups were performed using the Mann–Whitney *U*-test. Spearman’s correlation analysis was performed to evaluate the relationships between variables.

## Results

In the study, 58.6% (n = 41) of the participants were female, 41.6% (n = 29) were male. In the study, the average age was calculated as 26.14 ± 6.80. The average age of women was calculated as 26.68 ± 6.72, and the average age of men was calculated as 25.37 ± 6.95. Demographic information of the participants is given in Table 1.

The participants showed statistically significant enhancement in mean NOSE, ROE, and BAI points after surgery compared to preoperative values (*P* < .05) (Table 2).

The participants’ preoperative and postoperative SF-36 subscale scores were reported in Table 2; there were statistically significant distinctions in terms of physical role, physical function, general health, and social functionality perception values (*P* < .05) (Table 2).

Based on the preoperative BDI values, 70% (n = 49) of the patients were classified as normal, while 22.9% (n = 16) were mildly depressed, 5.7% (n = 4) were moderately depressed, and 1.4% (n = 1) were severely depressed. Postoperatively, 78.6% (n = 55) of the patients were classified as normal, 17.1% (n = 12) as mildly depressed, and 4.3% (n = 3) as moderately depressed.

The patients were divided into 2 groups according to their normal and higher depression levels. Group 1 included patients with BDI levels between 0-9, and Group 2 included patients with BDI scores of 10 and above. Seventy percent (n = 49) of the patients were in Group 1, and 30% (n = 21) were in Group 2. There were statistically significant distinctions in the mean values of preoperative BAI, emotional role, physical role, bodily pain, social function, and general health perception according to the depression status of the patients. There were statistically significant differences in the mean values of preoperative BAI, physical role, emotional role, social function, bodily pain, and general health perception according to the depression status of the preoperative participants (*P* < .05) (Table 3). There was a statistically significant change in the mean values of NOSE, ROE, BAE, physical function, physical role difficulty, and general health perception in Group 1 patients (*P* < .05) (Table 3). Statistically significant differences were found between the preoperative and postoperative mean scores of NOSE, ROE, physical function, physical role, and emotional role among Group 2 patients (*P* < .05) (Table 3).

Of the preoperative patients, 58.6% (n = 41) had minimal, 31.4% (n = 22) had mild, 5.7% (n = 4) had moderate, and 4.3% (n = 3) had severe anxiety. Postoperative patients had 62.9% (n = 44) minimal, 27.1% (n = 19) mild, 8.6% (n = 6) moderate, and 1.4% (n = 1) severe anxiety levels. Patients included in the study were divided into 2 groups according to their minimal and high anxiety levels. Statistically significant differences were observed in the mean preoperative emotional role, social function, and bodily pain scores among patients with differing anxiety statuses (*P* < .05) (Table 4).

There were statistically significant changes in the mean values of NOSE, ROE, physical function, and physical role in patients with low BAI values (*P* < .05) (Table 4).

The mean preoperative and postoperative NOSE, ROE, physical function, social function, and general health perception scores differed significantly in patients with high BAI values (*P* < .05) (Table 4).

Preoperative BDI scores showed a statistically significant positive correlation with postoperative NOSE scores (*P* < .05). Preoperative BDI scores showed a statistically significant negative correlation with postoperative ROE scores (*P* < .05). A high preoperative depression score negatively affected postoperative functional and aesthetic satisfaction (Table 5).

Preoperative BAI scores were not significantly associated with postoperative NOSE or ROE scores (*P* > .05). Preoperative anxiety levels did not affect postoperative functional or aesthetic satisfaction (Table 6).

## Discussion

Septorhinoplasty, a surgical procedure addressing both the cosmetic and functional aspects of the nose, has been increasingly performed in recent years and plays an important role in overall health. In recent years, psychological and social well-being has gained importance comparable to that of physical health. Consequently, quality-of-life scales used in the standard evaluation of disease and treatment effectiveness have come to the forefront. Quality of life is a broad concept encompassing physical, psychological, social, economic, cognitive, and sexual dimensions of life. General and disease-specific instruments are commonly used to assess quality of life.^1^^-^^4^

The present study evaluated the impact of septorhinoplasty on patients’ psychological status and well-being. For this purpose, the SF-36 as a general quality of life scale, NOSE and ROE scales were used, which are specific to septorhinoplasty. Significant improvements in physical function, social function, and general health perception parameters were observed in postoperative patients. It was found that NOSE and ROE scales were inversely related to each other and that there was a significant improvement in both scales postoperatively.

The BDI and the BAI were also used to evaluate the effects of septorhinoplasty on depression and anxiety. Postoperative anxiety levels showed a statistically significant improvement, whereas depression levels did not demonstrate a significant change.

This study demonstrates that preoperative depressive status significantly influences postoperative functional, aesthetic, and psychological outcomes following septorhinoplasty. Patients with low preoperative BDI scores showed better baseline quality-of-life parameters and lower anxiety levels, indicating a more favorable preoperative psychological profile.

Postoperatively, both low- and high-BDI groups exhibited significant improvements in ROE, physical functioning, and physical role scores, along with a significant decrease in NOSE scores, confirming the overall functional and aesthetic benefits of septorhinoplasty. However, psychological outcomes differed between groups. Patients with low BDI scores experienced a reduction in anxiety and an improvement in general health perception, whereas patients with high BDI scores demonstrated a significant decrease in depressive symptoms and improved emotional role functioning.

Ching et al^17^ demonstrated the validity and reliability of SF-36 and reported that its sensitivity was high in aesthetic surgery, particularly in rhinoplasty and breast reduction procedures. In the prospective study conducted by Ihvan et al,^18^ improvements in vitality, pain, general health, mental health, physical, and social functions (SF-36 subscales) were observed in patients who underwent septorhinoplasty.Similar results were obtained in this study as well.

Assessment of disease-related quality of life in nasal surgery relies on several condition-specific measurement instruments. Consistent with previous studies, the NOSE scale is a well-established and reliable instrument for the assessment of nasal obstruction.^19^^-^^21^ In the present study, significant improvements in postoperative NOSE scores further support the reliability and clinical usefulness of this scale in evaluating functional outcomes following septorhinoplasty. The ROE, which is specific to rhinoplasty, has been used in many studies and has been found to be associated with satisfaction with rhinoplasty.^19^^,^^22^

Küçür et al^23^ conducted the study with preoperative and healthy participants to assess the psychological well-being of patients scheduled for rhinoplasty. Statistically significant differences were found in the Liebowitz Social Anxiety Scale, but no differences were found in the Hospital Anxiety and Depression Scale and the Rosenberg Self-Esteem Scale in this study. Statistically meaningful differences were identified between the 2 groups with respect to the SF-36 domains of bodily pain, vitality, social functioning, and role emotional scores.^23^

Similar to the findings, previous studies have demonstrated that preoperative psychological status has a significant impact on postoperative satisfaction.^24^ In contrast, a study by Strazdins et al^25^ reported no association between mental well-being and patient-reported satisfaction.

Individuals seeking aesthetic rhinoplasty frequently demonstrate elevated social appearance–related anxiety and diminished self-esteem.^26^ Numerous studies have evaluated the psychological state of patients undergoing rhinoplasty.^26^^-^^30^ Naraghi et al^27^ compared depression levels in patients undergoing aesthetic and functional rhinoplasty; they found higher levels of depression in the aesthetic group. Belli et al^28^ also found that patients requesting cosmetic rhinoplasty had higher BDI and BAI levels. Yıldırım et al^29^ compared levels of depression and anxiety in patients who underwent septorhinoplasty and rhinoplasty and found that the septorhinoplasty group had higher levels of depression, whereas the rhinoplasty group exhibited higher levels of anxiety. Consistent with the findings, previous studies indicate that patients’ psychological status plays a significant role in postoperative satisfaction.^29^^,^^30^ Hohenberger et al^30^ showed that depression and anxiety negatively affect patient satisfaction in their study.

Köybaşı et al^31^ conducted a study to evaluate the effects of anxiety levels and functional outcomes on satisfaction in patients who underwent rhinoplasty in the last 2 years and found that functional outcomes were related to patient satisfaction. They found no correlation between anxiety and satisfaction. Hohenberger et al^32^ found that patients who underwent septorhinoplasty had higher depression and anxiety levels compared to the control group. High depression and anxiety levels negatively affected the postoperative quality of life scale.^32^

The findings suggest that septorhinoplasty promotes better psychological health and a higher overall quality of life. Specific life disturbances such as depression and anxiety require particular attention to the data of individuals undergoing septorhinoplasty. A consensus scale could be developed to determine whether septorhinoplasty improves the quality of life, and further studies should be conducted in this direction.

## Figures and Tables

**Table 1.** Demographic Distribution

	**n**	**Percent (%)**
Gender Female Male	41 29	58.6 41.4
Working status Working Not working	36 34	51.4 48.6
Marital status Marired Single	17 53	24.3 75.7
Education level Low Medium High	11 18 41	15.8 25.7 58.6
Age 18-25 26 and high	38 32	54.3 45.7

**Table 2.** Preoperative and Postoperative Comparison of NOSE, ROE, BDI, BAI, and Subscales of SF-36 Values of the Patients Included in the Study

	**Preop**			**Postop**			***P***
	**Median ** **(min-max)**	**IQR**	**Mean ± SD**	**Median ** **(min-max)**	**IQR**	**Mean ± SD**	
NOSE	60 (10-100)	31.25	60.6.4 ± 2379	20 (0-100)	30	24,87 ± 24,88	.000*
ROE	16.6 (0-66.66)	20.83	21.1 ± 15.9	70.83 (25-100)	34.38	68.40 ± 22.40	.000*
BDI	4 (0-35)	10.25	6.31 ± 7.26	3.5 (0-22)	8.25	5.31 ± 5.95	.177
BAI	6 (0-36)	8.25	7.75 ± 7.20	4 (0-29)	10	6.14 ± 6.36	.005*
Physical function	80 (0-100)	35	74.35 ± 24.90	92.5 (50-100)	92.5 (50-100)	88.50 ± 13.89	.00*
Physical role	100 (0-100)	56.25	70.00 ± 38.21	100 (0-100)	0	90.00 ± 23.07	.00*
Emotional role	10 (0-100)	66.67	67.13 ± 41.11	100 (0-100)	33.36	76.66 ± 35.14	.105
Vitality	50 (15-95)	16.25	49.85 ± 12.51	45 (25-100)	15	47.42 ± 11.42	.076
Emotional well-being	44 (0-75)	12	45.08 ± 10.28	44 (20-100)	8	45.74 ± 11.10	.95
Social function	51.25 (0-75)	50	75.50 ± 23.03	87.5 (37.5-100)	28.13	82.00 ± 18.00	.024*
Bodily pain	90 (0-100)	25	82.35 ± 21.53	87.5 (55-100)	22.5	87.00 ± 14.61	.098
General health	70 (20-100)	25	67.45 ± 16.37	75 (25-100)	30	73.02 ± 17.79	.005*

*Wilcoxon signed-rank test; *P* < .05 is significant

BAI, Beck Anxiety Inventory; BDI, Beck Depression Inventory; IQR, interquartile range; NOSE, Nasal Obstruction Symptoms Evaluation; max, maximum; min, minimum; Preop, preoperative; Postop, postoperative; ROE, Rhinoplasty Outcome Evaluation; SD, standard deviation; SF-36, Short Form-36.

**Table 3.** Preoperative and Postoperative Comparison of Mean Values of NOSE, ROE, BAI, and Subscales of SF-36 Quality of Life Scale According to Depression Status and BDI Levels of Patients Included in the Study

	**Preop** **Mean ± SD**		**Postop** **Mean ± SD**		**Low BDI Value ** **(0-9)** **n = 49** **Group 1**		**High BDI Value** **(10 and more)** **n = 21** **Group 2**	
	**Low BDI value** **n = 49** **Group 1**	**High BDI value** **n = 21** **Group 2**	**Low BDI** ** value** **n = 49** **Group 1**	**High BDI value** **n = 21** **Group 2**	**Preop** **Mean ± SD**	**Postop** **Mean ± SD**	**Preop** **Mean ± SD**	**Postop** **Mean ± SD**
NOSE	59.28 ± 24.20	63.80 ± 23.07	24.00 ± 27.20	26.90 ± 18.80	59.28 ± 24.2	24 ± 27.2	63.8 ± 23.07	26.9 ± 18.8
	*P* = .695		*P* = .171		*P* = .000*		*P* = .000*	
ROE	23.07 ± 17.39	16.63 ± 11.17	71.35 ± 22.57	61.50 ± 20.91	23.07 ± 17.39	71.35 ± 22.57	16.63 ± 11.17	61.50 ± 20.91
	*P* = .191		*P *= .09		***P* = .000** *		*P* = .000*	
BAI	6.44 ± 6.89	10.80 ± 7.12	4.97 ± 6.41	8.85 ± 5.45	6.44 ± 6.89	4.97 ± 6.41	10.80 ± 7.12	8.85 ± 5.45
	*P* = .006*		*P* = .004*		*P* = .013*		*P* = .197	
Physical function	75.10 ± 24.37	72.61 ± 26.62	89.79 ± 13.57	85.47 ± 14.48	75.10 ± 24.37	89.79 ± 13.57	72.61 ± 26.62	85.47 ± 14.48
	*P *= .656		*P *= .105		*P* = .00*		*P* = .017*	
Physical role	77.55 ± 34.32	52.38 ± 41.76	90.81 ± 22.64	88.09 ± 24.51	77.55 ± 34.32	90.81 ± 22.64	52.38 ± 41.76	88.09 ± 24.51
	*P* = .012*		*P* = .401		*P* = .007*		*P* = .006*	
Emotional role	76.86 ± 36.13	44.44 ± 43.88	81.63 ± 31.22	65.07 ± 41.46	76.86 ± 36.13	81.63 ± 31.22	44.44 ± 43.88	65.07 ± 41.46
	*P* = .003*		*P* = .07		*P* = .48		*P* = .002*	
Vitality	49.8 ± 12.68	49.76 ± 12.39	48.77 ± 11.94	44.28 ± 9.65	49.89 ± 12.68	48.77 ± 11.94	49.76 ± 12.39	44.28 ± 9.65
	*P* =.688		*P* = .144		*P* = .341		*P* = .07	
Emotional well-being	44.08 ± 10.04	47.42 ± 10.68	46.00 ± 11.47	45.14 ± 10.44	44.08 ± 10.04	46.00 ± 11.47	47.42 ± 10.68	45.14 ± 10.44
	*P* = .449		*P* = .406		*P* = .545		*P* = .252	
Social function	80.00 ± 22.67	65.00 ± 20.76	84.48 ± 17.56	76.195 ± 18.07	80 ± 22.67	84.48 ± 17.56	65 ± 20.76	76.19 ± 18.07
	*P* = .009*		*P *= .059		*P *= .124		*P* = .071	
Bodily pain	86.12 ± 19.14	73.57 ± 24.59	90.51 ± 14.03	78.80 ± 12.78	86.12 ± 19.14	90.51 ± 14.03	73.57 ± 24.59	78.80 ± 12.78
	***P* = .026**		***P* = .001**		*P* = .189		*P* = .354	
General health	71.46 ± 13.92	58.09 ± 18.12	78.10 ± 16.04	61.19 ± 16.27	71.46 ± 13.92	78.1 ± 16.04	58.09 ± 18.12	61.19 ± 16.27
	*P* = .003*		*P* = .00*		*P* = .01*		*P *= .274	

*Mann–Whitney *U*-test; *P* < .05 is significant

BAI, Beck Anxiety Inventory; BDI, Beck Depression Inventory; NOSE, Nasal Obstruction Symptoms Evaluation; Preop, preoperative; Postop, postoperative; ROE, Rhinoplasty Outcome Evaluation; SD, standard deviation; SF-36, Short Form-36.

**Table 4.** Preoperative and Postoperative Comparison of Mean Values of NOSE, ROE, BDI, and Subscales of SF-36 Quality of Life Scale According to Anxiety Status and Level of Patients Included in the Study

	**Preop** **Mean ± SD**		**Postop** **Mean ± SD**		**Low BAI value** **(0-9)** **n = 49**		**High BAI value** **(10 and more)** **n = 21**	
	**BAI** **Low**	**BAI** **High**	**BAI** **Low**	**BAI** **High**	**Preop** **Mean ± SD**	**Postop** **Mean ± SD**	**Preop** **Mean ± SD**	**Postop** **Mean ± SD**
NOSE	63.45 ± 26.63	56.66 ± 18.79	27.07 ± 30.29	21.75 ± 14.06	63.45 ± 26.63	27.07 ± 30.29	56.66 ± 18.79	21.75 ± 14.06
	*P *= .274		*P* = .632		*P *= .000*		*P* = .000*	
ROE	20.09 ± 16.71	22.52 ± 15.05	70.44 ± 21.78	65.51 ± 23.32	20.98 ± 16.71	70.44 ± 21.78	22.52 ± 15.05	65.51 ± 23.32
	*P *= .433		P = .315		*P *= .000*		*P* = .000*	
BDI	5.09 ± 7.22	8.03 ± 7.08	4.65 ± 5.71	6.24 ± 6.26	3.24 ± 2.46	3.43 ± 4.61	14.13 ± 6.86	9.96 ± 6.59
	*P* = .02*		*P* = .205		***P* = .616**		*P* = .00*	
Physical function	77.31 ± 25.42	70.17 ± 23.95	91.58 ± 12.72	84.13 ± 14.52	77.31 ± 25.42	91.58 ± 12.72	70.17 ± 23.95	84.13 ± 14.52
	*P *= .104		*P *= .005*		*P* = .00*		*P *= .007	
Physical role	76.82 ± 34.63	60.34 ± 41.46	94.51 ± 18.12	83.62 ± 27.77	76.82 ± 34.63	94.51 ± 18.12	60.34 ± 41.46	83.62 ± 27.77
	*P *= .09		*P *= .025*		*P* = .004*		*P *= .18	
Emotional role	77.23 ± 37.59	52.87 ± 42.27	82.1 ± 30.82	68.96 ± 39.77	77.23 ± 37.59	82.10 ± 30.82	52.87 ± 42.27	68.96 ± 39.77
	*P* = .01*		*P* = .19		*P* = .536		*P *= .113	
Vitality	50.48 ± 13.91	48.96 ± 10.38	47.65 ± 13.09	47.1 ± 8.74				
	*P *= .519		*P *= .81		*P *= .187		*P *= .235	
Emotional well-being	43.92 ± 10.52	46.72 ± 9.87	45.9 ± 11.33	45.51 ± 10.97	43.92 ± 10.52	45.90 ± 11.33	46.72 ± 9.87	45.51 ± 10.97
	*P *= .396		*P *= .98		*P *= .693		*P *= .582	
Social function	82.01 ± 23.39	66.29 ± 19.38	85.36 ± 16.51	77.24 ± 19.2	82.01 ± 23.39	85.36 ± 16.51	66.29 ± 19.38	77.24 ± 19.20
	*P *= .002*		*P *= .07		*P *= .328		*P *= .024	
Bodily pain	85.6 ± 22.6	77.75 ± 19.39	90.85 ± 13.58	81.55 ± 14.49	85.60 ± 22.60	90.85 ± 13.58	77.75 ± 19.39	81.55 ± 14.49
	*P* = .02*		*P* = .005*		*P *= .328		*P *= .313	
General health	70.04 ± 15.46	63.79 ± 17.19	74.02 ± 20.06	71.62 ± 14.19	70.04 ± 15.46	74.02 ± 20.06	63.79 ± 17.19	71.62 ± 17.19
	*P *= .245		*P *= .362		*P *= .078		*P *= .025	

*Mann–Whitney *U-*test; *P* < .05 is significant.

BAI, Beck Anxiety Inventory; BDI, Beck Depression Inventory; NOSE, Nasal Obstruction Symptoms Evaluation; Preop, preoperative; Postop, postoperative; ROE, Rhinoplasty Outcome Evaluation; SD, standard deviation; SF-36, Short Form-36.

**Table 5.** Correlation of Preoperative BDI Scores with Postoperative NOSE and ROE Scores of Patients Included in the Study

	**PREBDE**	**POSTNOSE**	**POSTROE**
**PREBDE, *r*** ***P***	1	0.305 .01*	−0.303 .011
**POSTNOSE, *r*** ***P***	—	1	−0.340 .004*
**POSTROE, *r*** ***P***	—	—	1

*Spearman’s correlation; <.05

BDI, Beck Depression Inventory; NOSE, Nasal Obstruction Symptoms Evaluation; Pre, preoperative; Post, postoperative; ROE, Rhinoplasty Outcome Evaluation; *r*, correlation coefficients.

**Table 6.** Correlation of Preoperative BAI Scores with Postoperative NOSE and ROE Scores of Patients Included in the Study

	**PREBAI**	**POSTNOSE**	**POSTROE**
**PREBAI, *r*** ***P***	1	.14 .247	−.178 .14
**POSTNOSE, *r*** ***P***	—	1	−.340 .004*
**POSTROE, *r*** ***P***	—	—	1

*Spearman’s correlation; <.05

BDI, Beck Depression Inventory; NOSE, Nasal Obstruction Symptoms Evaluation; Pre, preoperative; Post, postoperative; ROE, Rhinoplasty Outcome Evaluation; *r*, correlation coefficients.

## Data Availability

The data that support the findings of this study are available on request from the corresponding author.
